# Antibiotic overuse in a contemporary cohort of children hospitalized with influenza, RSV, or SARS-CoV-2: a retrospective cohort study

**DOI:** 10.1186/s12887-025-06165-8

**Published:** 2025-10-03

**Authors:** Mrinmoyee Kalasikam, Natalia Jimenez-Truque, Anisa B. Kloek, Ritu Banerjee

**Affiliations:** 1 University of Tennessee Health Science Center, Memphis, TN USA; 2 Vanderbilt Vaccine Research Program, Vanderbilt University Medical Center, Nashville, TN USA; 3 Occidental College, Los Angeles, CA USA; 4 Division of Pediatric Infectious Diseases, Vanderbilt University Medical Center, 1161 21 st Avenue, MCN A3101C, Nashville, TN 37232 USA

**Keywords:** Antibiotic overuse, Influenza, RSV, COVID-19, Viral respiratory tract infection

## Abstract

**Background:**

Children hospitalized with viral lower respiratory tract infections (LRTIs) are often prescribed antibiotics due to concern for bacterial co-infection, although most do not have concurrent bacterial infections. This unnecessary antibiotic treatment can lead to bacterial resistance and adverse events. The extent of antibiotic overuse in hospitalized children with community-onset viral LRTIs has not been described in recent years. To identify antibiotic stewardship opportunities in this population, we quantified the extent of antibiotic overtreatment and determined predictors of antibiotic use among children hospitalized with influenza, respiratory syncytial virus (RSV), or SARS-CoV-2 (COVID-19).

**Methods:**

We performed a single-center retrospective study evaluating antibiotic use and culture-confirmed bacterial co-infection among children and adolescents hospitalized with influenza, RSV, or COVID-19 between April 2020 and May 2023. Predictors of antibiotic treatment were determined using logistic regression.

**Results:**

We included 1,718 patients (influenza: 188; RSV: 1,022; COVID-19: 535). Patients with RSV were younger and more likely to be in intensive care. While only 8% of patients had culture-confirmed bacterial co-infection, the proportion receiving antibiotics was high and varied by virus (influenza: 60.6%, RSV:41.2%, COVID-19: 48.6%, *p* < 0.001). Independent predictors for receipt of > 3 days of antibiotics were elevated inflammatory markers, comorbidities, mechanical ventilation, intensive care unit admission, influenza infection, and a concurrent non-respiratory infection.

**Conclusions:**

In children hospitalized with community-onset viral LRTIs, antibiotic treatment is substantially higher than the burden of culture-confirmed bacterial infection, especially for influenza, suggesting antibiotic overuse and antibiotic stewardship opportunities.

**Supplementary Information:**

The online version contains supplementary material available at 10.1186/s12887-025-06165-8.

## Background

The burden of pediatric hospitalizations for viral lower respiratory tract infections (LRTIs) is substantial. In the U.S., over 22,000 children have been hospitalized with COVID-19 since 2020, 12,000–46,000 children are hospitalized with influenza each year, and up to 80,000 children < 5 years are hospitalized with RSV every year [1–3]. Children hospitalized with viral LRTIs are often treated with antibiotics due to concerns for bacterial co-infection, especially bacterial pneumonia, which can be preceded by viral infection ([4, 5] [6]). However, the majority of children with viral LRTIs do not have concurrent bacterial infections, so this practice leads to unnecessary antibiotic treatment which, in turn, can lead to development of resistant bacteria and adverse events. Unlike in adults, the extent of antibiotic overuse in hospitalized children with community-onset viral LRTI has not been well-quantified in recent years [4].

Several studies have found that although there were low rates of bacterial co-infection in adults hospitalized with COVID-19, up to 75% received antibiotics [7, 8]. Similarly, a large multicenter study evaluating children with critical illness from COVID-19 found that 7% of children had bacterial coinfection but 63% received antibiotics [5]. Studies of antibiotic treatment among children hospitalized with influenza or RSV before the COVID-19 pandemic demonstrated that 60–90% received antibiotics [9, 10], but there were few evaluations of bacterial co-infection rates [11–14]. Furthermore, most prior studies of viral LRTI do not quantify antibiotic use and may have overestimated bacterial co-infection burden because they interpreted any respiratory culture with bacterial growth as indicative of bacterial infection, although many positive respiratory cultures likely reflected colonization [15–17]. We sought to quantify the extent of antibiotic overuse in a contemporary cohort of children hospitalized with viral LRTI, in the context of an active hospital-based antibiotic stewardship program.

## Methods

### Sampling and exclusion criteria

We conducted a single center retrospective cohort study of all children and adolescents aged < 19 years old hospitalized at Monroe Carell Jr. Children’s Hospital at Vanderbilt, Nashville, TN, a quarternary care, 400 bed free-standing children’s hospital located in an urban area of the Southeastern United States. Included patients had a positive antigen or polymerase chain reaction (PCR) test for influenza, RSV, or COVID-19. Patients with RSV and influenza were hospitalized January 2021 to May 2023, and patients with COVID-19 were hospitalized April 2020 to February 2022. Patients were excluded if they were > 19 years of age or were not hospitalized.

From 2021 onwards, the institutional antibiotic stewardship team performed daily audit and feedback of antibiotics prescribed in inpatients; feedback was provided to treating providers of all admitting services by a stewardship physician or pharmacist via telephone or in person discussion, if indicated. Audit and feedback were performed inconsistently during 2020 due to disruptions from the COVID-19 pandemic. Viral testing was qualitative and results were available within 24 h of specimen collection. Molecular testing was not used for evaluation of pleural fluid or other respiratory specimens.

### Data collection

Medical records were reviewed to identify patients with positive tests for influenza, RSV, or COVID-19 within 48 h of hospitalization and to collect demographic and clinical characteristics including age, sex, race, microbiology results, hospital length of stay, laboratory results, and antibiotic administration in the Emergency Department or any inpatient hospital unit. We did not include antibiotics received at other hospitals or in the outpatient setting prior to admission. Race and ethnicity were both self-reported. Culture-confirmed bacterial infection was defined as growth of clinically significant bacteria (non-commensal species) indicative of bacterial co-infection from any specimen source including blood, urine, pleural fluid, cerebrospinal fluid, and respiratory secretions (sputum, tracheal aspirates) throughout the hospitalization. Time of infection detection was defined as time bacterial growth from clinical specimens was reported in the electronic medical record. We also used a second, more stringent definition of culture-confirmed bacterial infection that excluded cultures of respiratory specimens. We did not collect information on presence of white blood cells (WBC) in respiratory cultures. Cultures from any specimen type with growth of common commensals, including diphtheroids, *Micrococcus* species, *S. viridans*, and coagulase-negative *Staphylococci* were considered colonizers and not bacterial infection. Patients with multiple viruses were excluded from multivariable logistic regression models. Comorbidities, symptomatic respiratory tract infection, and concurrent non-respiratory infections were assigned if an appropriate ICD-10 code was associated as primary or other diagnosis during a hospital encounter (Supplemental Table).

### Data analysis

After excluding patients with detection of multiple viruses, two different multivariable logistic regression models were developed a priori to identify predictors of (1) at least one dose of antibiotic treatment and (2) greater than 3 days of antibiotic treatment. The model included variables that were deemed clinically significant based on clinical judgement.The logistic regression models included the type of virus, age, presence of elevated inflammatory markers [procalcitonin, PCT, (≥ 0.5 ug/mL) or C-reactive protein, CRP (≥ 50 mg/L)], intensive care unit (ICU) status, comorbidities, concurrent non-respiratory infection and whether the patient received mechanical ventilation. In the final multivariable models, patients who did not have inflammatory markers measured were assumed to have normal inflammatory markers; results did not differ when we ran the models including only patients with PCT or CRP results. Bacterial co-infection and antibiotic utilization percentages were determined by type of virus. Analyses were done using Stata 17 (Stata Corp BE).

## Results

### Demographic and clinical characteristics (Table 1)

**Table 1:** Demographic and clinical characteristics of pediatric patients by type of virus

Characteristic	Influenza (*N* = 188; 10.9%)^a^	RSV (*N* = 1,022; 59.5%)^b^	COVID-19 (*N* = 535; 31.1%)^c^	Total (*N* = 1,718)^d, e^
Demographics				
Median age in years (IQR)	5.2 (2.2–9.9)	0.6 (0.2 −2.0)	6.1 (0.8–14.6)	1.3 (0.3–5.6)
Neonates (< 30 days of life)	5 (2.7%)	109 (10.7%)	35 (6.5%)	148 (8.6%)
Male sex	108 (57.4%)	547 (53.5%)	278 (52.0%)	922 (53.7%)
Race				
Asian	5 (2.7%)	6 (0.6%)	3 (0.6%)	14 (0.8%)
Black or African American	27 (14.4%)	142 (13.9%)	114 (21.3%)	279 (16.2%)
White	126 (67.0%)	673 (65.9%)	320 (59.8%)	1,104 (64.3%)
Other Race/Unknown	30 (16.0%)	201 (19.7%)	98 (18.3%)	321 (18.7%)
Ethnicity				
Hispanic or Latino	42 (22.3%)	185 (18.1%)	100 (18.7%)	320 (18.6%)
Not Hispanic or Latino	81 (43.1%)	345 (33.8%)	150 (28.2%)	572 (33.3%)
Unknown	65 (34.6%)	492 (48.1%)	285 (53.3%)	826 (48.1%)
LOS, median days (IQR	2.7 (1.6 −5.0)	2.8 (1.7–4.8)	2.6 (1.3–5.3)	2.7 (1.6–4.9)
Severity				
ICU	55 (29.3%)	462 (45.2%)	117 (21.9%)	618 (36.0%)
Mechanical Ventilation	23 (12.2%)	108 (10.6%)	63 (11.8%)	187 (10.9%)
ECMO	2 (1.1%)	4 (0.39%)	4 (0.75%)	10 (0.58%)
Death	3 (1.6%)	16 (1.6%)	31 (6.2%)	48 (2.8%)
Symptomatic Respiratory Infection^f^	124 (66.0%)	848 (83.0%)	359 (67.1%)	1,305 (75.6%)
Non-respiratory infection^g^	10 (5.3%)	40 (3.9%)	27 (5.1%)	76 (4.4%)
Comorbidities	70 (37.2%)	169 (16.5%)	172 (27.1%)	380 (22.1%)
Cardiac Disease	2 (1.1%)	22 (2.2%)	15 (2.8%)	38 (2.2%)
Multiple conditions	2 (1.1%)	16 (1.6%)	8 (1.5%)	25 (1.5%)
Craniofacial condition	1 (0.5%)	3 (0.3%)	2 (0.4%)	6 (0.4%)
Diabetes Mellitus	8 (4.3%)	2 (0.2%)	15 (2.8%)	25 (1.5%)
Genetic Syndrome	3 (1.5%)	13 (1.3%)	8 (1.5%)	24 (1.4%)
Sickle Cell Disease	8 (4.3%)	3 (0.3%)	14 (2.6%)	25 (1.5%)
Immunocompromise^h^	17 (9.0%)	16 (1.6%)	39 (7.3%)	72 (4.2%)
Liver disease	0	5 (0.5%)	4 (0.8)	9 (0.5%)
Neurologic Disease	8 (4.3%)	18 (1.8%)	24 (4.5%)	50 (2.9%)
Prematurity	0	13 (1.3%)	1 (0.2%)	13 (0.8%)
Renal Disease	2 (1.1%)	6 (0.6%)	3 (0.6%)	11 (0.6%)
Asthma	19 (10.1%)	52 (5.1%)	12 (2.2%)	82 (4.8%)
Microbiology				
Bacterial Infection	15 (8.0%)	70 (6.9%)	55 (10.3%)	138 (8.0%)
Blood	5 (2.7%)	23 (2.3%)	18 (3.4%)	45 (2.6%)
Respiratory secretions	8 (4.3%)	34 (3.3%)	16 (3.0%)	57 (3.3%)
Urine	1 (0.5%)	11 (1.1%)	18 (3.4%)	30 (1.7%)
Other body fluid	1 (0.5%)	2 (0.2%)	2 (0.4%)	5 (0.3%)
Other	0	0	1 (0.2%)	1 (0.1%)^i^
Inflammatory Markers				
Inflammatory markers (PCT or CRP) measured	87 (46.3%)	305 (29.8%)	296 (55.3%)	671 (39.1%)
Elevated inflammatory markers [PCT (≥ 0.5 ug/mL) or CRP (≥ 50 mg/L)]^j^	44 (50.6%)	124 (40.7%)	102 (34.5%)	264 (39.3%)
PCT, median (IQR) [*n* = 435]	0.6 (0.1–2.6)	0.3 (0.1–1.4)	0.2 (0.1–0.6)	0.2 (0.1–1.1)
CRP, median (IQR) [*n* = 388]	38.7 (9.1–90.0)	32.7 (9.4–74.6)	24.7 (6.3–71.9)	30.9 (6.9–78.5)
Antibiotic Treatment				
Received any antibiotics	114 (60.6%)	421 (41.2%)	260 (48.6%)	785 (45.7%)
Duration of antibiotics				
No antibiotics	74 (39.4%)	601 (58.8%)	275 (51.4%)	933 (54.3%)
1–3 days	42 (22.3%)	185 (18.1%)	109 (20.4%)	331 (19.3%)
> 3 days	72 (38.3%)	236 (23.1%)	151 (28.2%)	454 (26.4%)
Antibiotics DOT median (IQR) [*n* = 785]	5 (3–8)	4 (2–7)	5 (2–10)	4 (2–8)

*IQR* Interquartile range, *LOS* Length of stay, *ECMO* Extracorporeal membrane oxygenation, *PCT* Procalcitonin, *CRP* C-reactive protein, *DOT* Days of therapy

^a^ Hospital admission from 12/20/2020–05/27/2023

^b^ Hospital admission from 04/01/2021–05/03/2023

^c^ Hospital admission from 04/06/2020-2/14/2022

^d^ Hospital admission from 04/06/2020–05/27/2023

^e^ 6 participants had influenza and RSV coinfection; 3 had influenza and COVID-19; 17 had RSV and COVID-19

^f^ ICD-10 diagnosis codes found in Supplemental Table 1

^g^Includes: acute otitis media (*N* = 32), mastoiditis (*N* = 1), abscess (*N* = 9), appendicitis (*N* = 9), cellulitis/skin infection (*N* = 11), cystitis ((*N* = 2), endopthalmitis (*N* = 1), Group A streptococcal pharyngitis (*N* = 3), meningitis (*N* = 5), pancreatitis (*N* = 2), endocarditis

^h^Includes patients with history of oncologic disease, solid organ or stem cell transplant, neutropenia, or primary immunodeficiency

^i^Cultured from a wound

^j^Percent of those with PCT or CRP obtained

We included 1,718 pediatric patients (188 with influenza, 1,022 with RSV, 535 with COVID-19). Patient demographic and clinical factors varied by virus type (Table 1). Median age was lowest for patients with RSV and highest for patients with COVID-19 (0.61 years vs. 6 years). Racial distribution varied by virus.A greater proportion of patients with RSV (45%) were in the ICU than patients with influenza or COVID-19. Mortality was highest in patients with COVID-19 at 6.2% but was lower for patients with influenza or RSV. Inflammatory markers among patients in whom they were measured, were elevated in almost half of patients with influenza, and in lower percentages of patients with RSV and COVID-19. The overall rate of bacterial co-infection was 8% when including respiratory cultures and 5.4% when excluding respiratory cultures. Positive cultures (of all specimen types including respiratory) occurred more commonly in patients with COVID-19 (10.3%) than those with RSV (6.9%) or influenza (8%). The median number of days from admission to detection of a positive bacterial culture was 3 (IQR 3 to 5) and did not vary by virus type.

### Antibiotic treatment

**Fig. 1:**
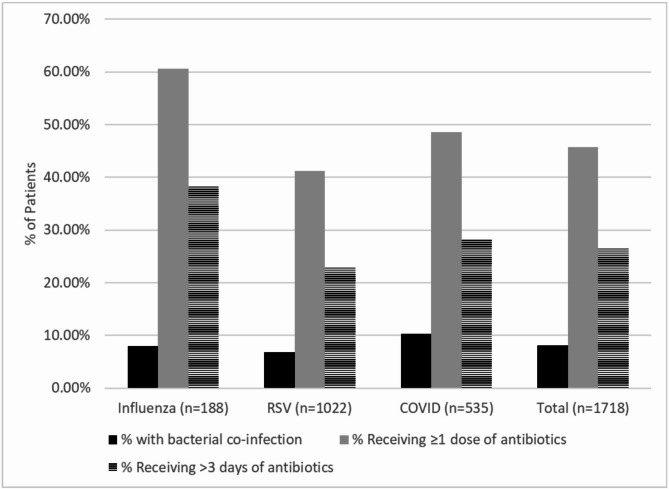
Culture-confirmed bacterial co-infection and antibiotic utilization among patients hospitalized with influenza, RSV, and COVID-19. Black, percentage of patients who had a culture-confirmed bacterial infection, including respiratory specimens; gray, percentage of patients who received at least one dose of antibiotic; horizontal lines, percentage of patients who received > 3 days of antibiotics

Although few patients had culture-confirmed bacterial infections, 45.7% of patients received at least one dose of antibiotics and 26.4% received > 3 days of antibiotics (Fig. 1). Antibiotic treatment varied by virus type. Among patients with influenza, 60.6% received at least one antibiotic dose, and 38% received > 3 days of antibiotics. In contrast, only 23% of patients with RSV and 28% of patients with COVID-19 received > 3 days of antibiotics (*p* < 0.001). Among a subgroup of patients with low severity of illness (not admitted to the ICU and with normal inflammatory markers), 7.3% had culture-confirmed bacterial infection including respiratory cultures, while 48.1% received at least 1 dose of antibiotic and 20.8% received > 3 days of antibiotics; these percentages did not differ significantly by virus type. In the multivariable logistic regression model, independent predictors of receiving > 3 days of antibiotic treatment were elevated inflammatory markers, being on mechanical ventilation, being in the ICU, having any comorbidity, and having a concurrent non-respiratory infection or influenza (Table 2). The same variables were independent predictors for receiving at least 1 dose of antibiotics.

**Table 2:** Multivariable analysis to determine predictors of receiving > 3 days of antibiotic therapy

Characteristic	Odds Ratio	95% CI	*P*-Value
Age (years)	0.99	0.97–1.02	0.620
Elevated CRP or PCT^b, c^	4.64	3.38–6.39	< 0.001
Mechanical Ventilation^c^	6.38	4.13–9.86	< 0.001
ICU^c^	1.41	1.04–1.92	0.028
Virus			
RSV	Reference		
Influenza	1.84	1.23–2.76	0.003
COVID	1.17	0.84–1.64	0.343
Any comorbidity^c^	3.14	2.35–4.21	< 0.001
Non-respiratory infection^c^	4.96	2.94–8.37	< 0.001

^a^ The model included 1,691 observations; excludes patients with multiple viruses

^b^ CRP (≥ 50 mg/L) or PCT (≥ 0.5 ug/L)

^c^Binary variables; reference category not listed

## Discussion

In this large contemporary cohort of children hospitalized with influenza, RSV, or COVID-19, we found that the percent of patients with detected bacterial co-infection was low, while the percent of patients receiving antibiotic treatment was high. Overall, the proportion of patients prescribed antibiotics for > 3 days was 46%, which is 4–6 times greater than the proportion that had culture-confirmed bacterial infections. We observed that the proportion of patients receiving antibiotics was highest for patients with influenza (> 60%) and lowest for patients with RSV (41%). Notably, even among patients with the lowest severity of illness (not in the ICU and with normal inflammatory markers), in whom bacterial infection was least likely, any antibiotic treatment was administered to 48.1% of patients and > 3 days of antibiotics were given to 20.8% of patients, suggesting antibiotic overtreatment. The rates of antibiotic use seen in our study are similar to those reported in earlier studies, suggesting that antibiotic prescribing practices have not changed in recent years despite greater provider and public awareness of the importance of antibiotic stewardship [9, 10, 18–20].

While the low bacterial co-infection rate we observed is supported by recent adult and pediatric studies of patients with COVID-19, it contrasts with some earlier studies of influenza and RSV that reported higher bacterial co-infection rates of 27–40% [15–17]. This discrepancy may be due to fact that some earlier studies defined bacterial co-infection largely based on positive bacterial culture results from non-sterile sites such as sputum or respiratory secretions and used bacterial colony count thresholds that are not validated for diagnosis of bacterial pneumonia [15–17, 21]. Additionally, these studies found that *S. pneumoniae* and *S. aureus* were the most commonly isolated bacterial pathogens among children with viral LRTI [16, 17]. Though growth of these organisms from respiratory specimens may have reflected bacterial co-infection in some patients, they may have reflected colonization of the respiratory tract in others and thus may have overestimated bacterial co-infection rates [22].

Patient demographic and clinical factors differed by virus type, which may have influenced medical management and could explain why antibiotic treatment differed by virus. Differences in patient demographics and comorbidities may also have contributed to the higher mortality observed in patients infected with COVID-19 compared to those infected with influenza and RSV. Hospitalized patients with RSV were significantly younger and less likely to receive antibiotics than patients with influenza. Clinicians may have been more likely to avoid antibiotics in patients hospitalized with RSV because these children were most commonly infants with bronchiolitis and were not as acutely ill as the patients with influenza.

We determined that several variables were independent predictors for receipt of antibiotic treatment, including mechanical ventilation, being in the ICU, and having influenza, comorbidities, concurrent non-respiratory infection, or elevated inflammatory markers. Antibiotic treatment of patients with influenza may stem from widespread clinician awareness of reports of influenza-associated Gram-positive bacterial pneumonia and the associated high mortality [23–25]. Mechanical ventilation, ICU admission and comorbidities are predictors of antibiotic use likely because they reflect severe illness and medical complexity, respectively, in which empiric antibiotic treatment may be warranted. Similarly, antibiotic treatment is warranted for patients with concurrent infections that may not be culture-confirmed, which in this cohort was most commonly acute otitis media. However, antibiotic de-escalation in terms of narrowing spectrum or shortening duration of therapy in such patients is still possible and should be targets of stewardship programs. High inflammatory markers are likely strongly associated with antibiotic treatment because of clinician confidence that these tests indicate bacterial infection. However, these tests, especially CRP, have low specificity and low positive predictive value (PPV) for bacterial infection and can be elevated in viral infections [26]. Providers should be wary about prescribing antibiotics based solely on the results of inflammatory markers, without consideration of the clinical context.

Strengths of our study are the large sample size, use of a stringent definition of bacterial co-infection, and evaluation of a contemporary cohort. Our study also has limitations, including its single center, retrospective nature, which may reduce its generalizability. Additionally, because bacterial pneumonia is not often confirmed by culture and we did not collect information about abnormal chest radiographs, we may have underestimated the occurrence of bacterial pneumonia. While it is unlikely that almost half of patients (the proportion who received antibiotics in our cohort) developed bacterial pneumonia after LRTI, we did not use sensitive molecular diagnostics like multiplex PCR or next generation sequencing technologies to rule out presence of bacterial pathogens [27]. Additional limitations are that we identified subjects based on positive viral test results, determined symptomatic respiratory tract infection based on diagnosis codes, did not collect data on subjects with negative bacterial cultures, and did not confirm presence of respiratory symptoms through chart review. We utilized diagnostic codes to determine when antibiotic treatment was given for infections other than pneumonia (e.g. acute otitis media, appendicitis), which may not have ascertained all such cases. In addition, antimicrobial stewardship interventions were inconsistently implemented throughout 2020 due to the COVID-19 pandemic.

## Conclusions

We demonstrate in a contemporary cohort that unnecessary antibiotic use persists in hospital settings, as the antibiotic treatment burden far exceeds bacterial co-infections for children hospitalized with influenza, RSV, or COVID-19. Antibiotic stewardship programs should encourage clinicians to withhold or promptly deescalate antibiotics, particularly for children hospitalized with mild viral LRTI.

## Supplementary Information

Supplementary Material 1.

## Data Availability

The datasets generated and/or analysed during the current study include protected health information and are not publicly available per policy of Vanderbilt University Medical Center but a deidentified dataset is available from the corresponding author on reasonable request.
