# Supplementary material for: Antibiotic overuse in a contemporary cohort of children hospitalized with influenza, RSV, or SARS-CoV-2: a retrospective cohort study
Source: BMC Pediatr. 2025 Oct 3;25:759. doi: 10.1186/s12887-025-06165-8 (PMC12495774; doi:10.1186/s12887-025-06165-8)
Supplement: Supplementary file 1 — Supplementary Material 1. [file 12887_2025_6165_MOESM1_ESM.docx]

**Supplemental Table 1: ICD-10 diagnosis codes for symptomatic respiratory infection**

| **Diagnosis** | **ICD-10 Code** |
| --- | --- |
| Acute bronchiolitis due to RSV | J21.0 |
| Acute bronchiolitis, unspecified | J21.9 |
| Acute obstructive laryngitis | J05.0 |
| Acute respiratory distress | R06.03 |
| Acute respiratory failure with hypercapnia | J96.02 |
| Acute upper respiratory infection, unspecified | J06.9 |
| Adenovirus infection | B34.0 |
| Apnea | R06.81, G47.33, G47.31 |
| Cough, unspecified | R05.9 |
| COVID-19 | U07.1 |
| Hypoxemia | R09.02 |
| Influenza | J11.1, J10.1 |
| Pleural effusion | J91.8, J90 |
| Pneumonia | J18.9, J12.82 |
| Respiratory failure with hypoxia | J96.01 |
| Respiratory failure, unspecified | J96.00, J96.90 |
| Sepsis, unspecified | A41.9 |
| Shortness of breath | R06.02 |
| Symptoms and signs involving circulatory and respiratory systems | R09.89 |
| Tachycardia | R00.0 |
| Tachypnea | R06.82 |
| Viral infection, unspecified | B34.8, B34.9 |
| Viral pneumonia | J12.9 |
